# Effect of Uniaxial Compression Frequency on Osteogenic Cell Responses in Dynamic 3D Cultures

**DOI:** 10.3390/bioengineering10050532

**Published:** 2023-04-27

**Authors:** Georgia-Ioanna Kontogianni, Konstantinos Loukelis, Amedeo Franco Bonatti, Elisa Batoni, Carmelo De Maria, Raasti Naseem, Kenneth Dalgarno, Giovanni Vozzi, David B. MacManus, Subrata Mondal, Nicholas Dunne, Chiara Vitale-Brovarone, Maria Chatzinikolaidou

**Affiliations:** 1 Department of Materials Science and Technology, University of Crete, 70013 Heraklion, Greece; 2 Research Center E. Piaggio and Department of Information Engineering, University of Pisa, 56126 Pisa, Italy; 3 School of Engineering, Newcastle University, Newcastle upon Tyne NE1 7RU, UK; 4 School of Mechanical & Manufacturing Engineering, Dublin City University, D09 W6F4 Dublin, Ireland; 5 Department of Applied Science and Technology, Politecnico di Torino, 10129 Turin, Italy; 6 Foundation for Research and Technology Hellas (FORTH)-IESL, 70013 Heraklion, Greece

**Keywords:** dynamic cell culture, mechanical stimulation, uniaxial cyclic compression, bioreactor, 3D scaffold, osteogenic differentiation, bone formation, PLLA, PCL, PHBV

## Abstract

The application of mechanical stimulation on bone tissue engineering constructs aims to mimic the native dynamic nature of bone. Although many attempts have been made to evaluate the effect of applied mechanical stimuli on osteogenic differentiation, the conditions that govern this process have not yet been fully explored. In this study, pre-osteoblastic cells were seeded on PLLA/PCL/PHBV (90/5/5 wt.%) polymeric blend scaffolds. The constructs were subjected every day to cyclic uniaxial compression for 40 min at a displacement of 400 μm, using three frequency values, 0.5, 1, and 1.5 Hz, for up to 21 days, and their osteogenic response was compared to that of static cultures. Finite element simulation was performed to validate the scaffold design and the loading direction, and to assure that cells inside the scaffolds would be subjected to significant levels of strain during stimulation. None of the applied loading conditions negatively affected the cell viability. The alkaline phosphatase activity data indicated significantly higher values at all dynamic conditions compared to the static ones at day 7, with the highest response being observed at 0.5 Hz. Collagen and calcium production were significantly increased compared to static controls. These results indicate that all of the examined frequencies substantially promoted the osteogenic capacity.

## 1. Introduction

Bone is a highly dynamic tissue that undergoes continuous remodeling throughout the human lifetime based on the demand to adapt to the mechanical forces applied to bone tissue physiologically [1]. The skeleton supports the movement of the body, and mechanical forces are essential for the maintenance of the bone remodeling equilibrium [2], while the loss of mechanical stimulation can change the bone structure and increase fragility [3]. The mechanisms involved in sensing and translating mechanical forces in the body are an area of growing interest, and their elucidation will help to better understand bone disorders and pathologies, and to create new strategies for bone renewal [3].

Bone tissue engineering (BTE) aims to combine principles of engineering and life sciences to create constructs with specific structural, biological, and mechanical characteristics which are reflective of native tissue conditions [4]. Many attempts at BTE aim to mimic the dynamic nature of bone tissue with the application of different external mechanical forces to come one step closer to the native conditions [5,6]. Between the two main categories of polymers which are mostly employed for the fabrication of scaffolds in BTE, natural and synthetic, the natural-derived polymers can usually accommodate a suitable bioactive environment without depicting strong immunogenic effects [7]. Despite their great biological response, they usually lack the necessary mechanical stiffness required for the fabrication of BTE scaffolds [8]. Conversely, the use of synthetic-derived biopolymers is often favored when attempting to replicate mechanically hard tissues, due to their rigidity and high mechanical strength [9]. Such constructs can comprise, among others, biodegradable synthetic polymers, such as poly (lactic acid) (PLA) [10], poly(ε-caprolactone) (PCL) [11], poly(methyl methacrylate) (PMMA) [12], poly(3-hydroxybutyrate-co-3-hydroxyvalerate) (PHBV), or their combinations [13].

During the last few decades, the application of additive manufacturing (AM) technologies for the fabrication of 3D scaffolds has replaced the conventional technologies for the production of BTE scaffolds due to their ability to convert complex digital 3D designs into physical structures with the desired micro and macro environment [14]. Among the different AM technologies that are available, Fused Deposition Modeling (FDM) can process materials with mechanical strength relatable to that of native bone tissue [15]. As such, the fabrication of scaffolds which exhibit a tailored architecture can provide prime spatiotemporal conditions that can support cell survival, growth, and proliferation [16,17].

Static culture conditions are the established methodology to evaluate the biological responses of tissue engineering systems at the interface between cells and biomaterials; however, they have several limitations. One of the main drawbacks is the decreased supply of nutrients into large scaffolds due to insufficient levels of diffusion [18]. Moreover, static conditions do not take into account the impact of various mechanical stimuli on the cellular response, which has been found to exert a significant influence on the development and remodeling processes of the native bone tissue [1,19]. Understanding how cells sense and translate the applied forces is gaining growing interest in the research community [20]. The mechanical stimuli applied to the cells are recognized as regulators of the cell fate and functionality, since cells sense and transmit them to their interior or to other cells, and translate them to biochemical signals that affect their cellular responses [21]. These processes of mechanotransduction within bone cells have been reported to be affected by the characteristics of the in vivo bone environment, including the 3D lacunocanalicular network architecture and extracellular matrix composition [22]. The cells sense the applied force by specific molecules or protein complexes called mechanosensors. The identified mechanosensors in bone forming osteoblasts include ion channels, gap junctions, integrins, actin cytoskeleton, and guanine nucleotide-binding G-protein-coupled receptors (GPCRs), which, upon their mechanical stimulation, drive the activation of different intracellular pathways [23]. Osteoblast stimulation enhances the production of adenosine triphosphate (ATP), which activates the receptors of the neighboring cells to produce calcium; therefore, a gap-junction influx of extracellular calcium occurs [24]. This process activates a protein cascade and causes the production of alkaline phosphatase (ALP), prostaglandin E2, osteopontin, osteocalcin, nitric oxide, matrix metalloproteinase, extracellular signal-regulated kinase ERK1 and ERK2, and mitogen-activated protein kinase [25,26,27], and downregulates sclerostin expression [28].

Different groups have examined the effects of varying mechanical stimuli on cellular and biological responses, including hydrostatic compression [29], fluid flow shear stress [30], and substrate strain [31,32], aiming to replicate the dynamic conditions that exist in the native bone under an in vitro environment by exposing tissue engineered constructs to different external mechanical stimuli to assess their effect on long term bone development [5,6,33,34,35]. The dynamic culture of bone cells has been found to enhance cell proliferation [36] and differentiation [37]. Specifically, it has been reported that the application of flow perfusion on scaffolds fabricated from starch either with ethylene vinyl alcohol or with PCL caused an increase in calcium deposition from marrow stromal cells after 15 days in culture [38]. Another study showed that a microfluidic system with a precisely controlled flow not only enhanced the osteogenic differentiation of pre-osteoblastic MC3T3-E1 cells, but also affected their orientation along the direction of the flow [39].

Compression loading is another type of mechanical stimulation that aims to mimic the intricate mechanotransduction conditions that exist in the native bone [40]. For example, the adaptation to the applied mechanical stimuli depends on the range of the mechanical forces and the corresponding strain. A displacement range between 50 μm and 1500 μm was first described by Frost [41] as being associated with bone remodeling. Above this upper limit, bone tissue will begin to regrow as a counter measure against the local deformation it is being subjected to, while, below the lowest limit, the resorption process is mostly favored [41]. Additionally, Rubin and Lanyon suggested that an important aspect of the mechanical stimulation is the number of applied load cycles, which affects the bone structural adaptation in vivo. In greater detail, they showed that 4 cycles of loading maintained the cortical bone mass of turkey ulna, while more than 36 cycles increased the bone mass [42]. Some groups have also investigated how the intertwining of both frequency and strain parameters can affect the remodeling process in various in vivo conditions. Eickhoff et al. found that the bone tissue reacts to high frequencies [43], while Rubin et al. proved that high frequencies of 20 Hz induced a more effective pattern in the maintenance and hierarchical deposition of bone mass in comparison to lower frequencies of 1 Hz with the same levels of applied displacement (i.e., 100 μm) [44]. Similarly, another study showed that the proliferation of human-derived osteoblasts is affected by the applied cycle number and strain at a constant frequency and by the frequency at a constant number of applied cycles [45], with the condition at 1 Hz being the optimal frequency for cell proliferation. Studies have shown that osteoblasts’ differentiation and proliferation potential can be amplified after the application of both uniaxial and biaxial strain [45,46]; however, the understanding of the biochemical interactions that are involved in this process is still limited.

Although the existing literature has already furthered our understanding of the effect of dynamic culture conditions in BTE, there is still a lack of correlation between the duration, level of displacement, and the frequency of the applied loading regarding the bone formation process. This work aims to correlate specific parameters of mechanical stimulation, including frequency values of physiological body movement, with the osteogenic response of cells seeded in 3D printed scaffolds. To this end, this study evaluates the responses of pre-osteoblasts loaded into polymeric blend porous scaffolds consisting of PLLA/PCL/PHBV (90/5/5 wt.%), under both dynamic and static conditions, for 21 days. For the dynamic conditions, a strain of 8% (corresponding to a displacement of 400 μm, given the height of our scaffold) was subjected to three frequencies of mechanical stimulation (i.e., 0.5, 1, and 1.5 Hz) for 1 h daily, to determine their impact on the pre-osteoblastic maturation. Cell proliferation and morphology were monitored via a reduction-based cytotoxicity assay and scanning electron microscopy (SEM), respectively. In addition, the measurement of the ALP activity, and collagen and calcium production, were conducted for the determination of the osteogenic effect of the mechanical stimulation compared to the static culture conditions.

## 2. Materials and Methods

### 2.1. Overview of the Scaffold Design

**Figure 1:**
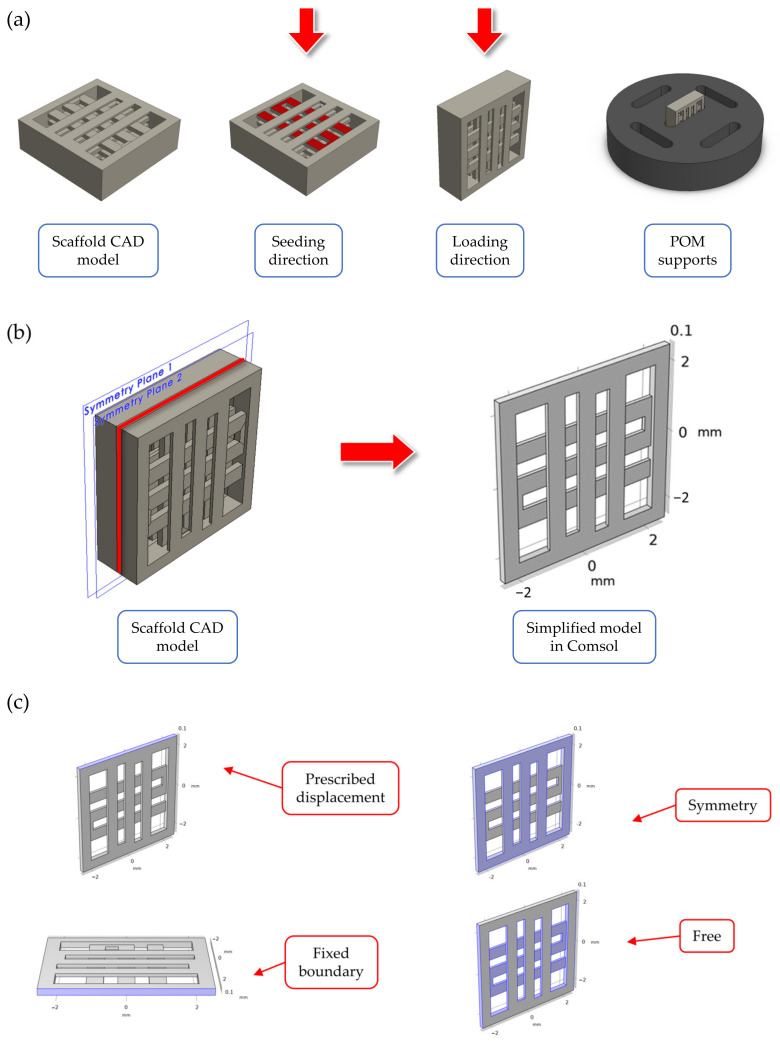
Summary of the scaffold design. In (**a**), an overview of: (i) the scaffold CAD model, (ii) the two directions for seeding and mechanical stimulation (indicated by a red arrow in the figure), and (iii) the CAD model for the custom CNC-milled POM support used to stabilize the scaffolds during stimulation. In (**b**), the simplified model through symmetry conditions for the mechanical loading FE simulations. In (**c**), a summary of the boundary conditions used in the FE models.

Computer Aided Design (CAD) model for the scaffold geometry was defined in Solidworks^®^ 2020 (Dassault Systeme, Velizy-Villacoublay, France), as can be seen in Figure 1a. The final geometry was selected from among another scaffold geometries following the results of preliminary FE simulations. The details about these simulations are reported in Supplementary Materials—Section S1. The selected geometry corresponds to a cubic scaffold with 5 mm sides and 1 mm height, sliced with 0.2 mm layer height, one full layer at the bottom and 50% internal porosity with 90° alternating infill line angle for the other layers. The first full layer was introduced in the geometry to encapsulate the cells inside the scaffolds and prevent them from slipping away during the cell seeding stage. To improve the strain transferred to cells during mechanical stimulation, the cell seeding direction (i.e., scaffold in horizontal configuration) was different than the one used for applying the displacement (i.e., scaffold in vertical configuration), as can be seen in Figure 1a. Custom supports were CNC milled using POM to keep the scaffolds stable during stimulation (Figure 1a). No lubricants were used during the support fabrication to avoid any contamination of the seeded scaffolds.

### 2.2. Finite Element Simulations of the Scaffold during Mechanical Compression

Finite element (FE) simulations were performed in COMSOL Multiphysics v6.0 (COMSOL AB, Stockholm, Sweden) using the solid mechanics module to investigate the displacement field and strain distribution inside the printed scaffolds during mechanical compression. The simulations were important to validate the scaffold design and loading arrangement, as well as to assure that cells seeded inside the scaffold experienced an appreciable level of strain during mechanical stimulation. Briefly, to reduce the model’s computational complexity, a simplified scaffold geometry was considered during the simulations (Figure 1b), consisting of two half layers (height equal to 0.1 mm each). This simplified geometry corresponded to a ‘slice’ of the scaffold center, while the presence of the other layers was modelled using symmetry boundary conditions (Figure 1c). The material properties for the FE model are reported in Table 1, and they refer to the PLLA/PCL/PHBV (90/5/5 wt.%) polymer blend used for scaffold fabrication (data presented in a previous paper using this material formulation [47]. The mechanical properties of the filament material after printing were determined based on the experimental data published in a previous paper by our group [48]. A linear, elastic, homogeneous, and isotropic behavior was considered for the material constitutive equations.

Regarding the boundary conditions, a prescribed displacement of 400 µm (equal to 8% strain of the scaffold side) was chosen for the loaded boundary to model the mechanical loading phase during in vitro stimulation, while the opposite boundary was set to fixed constraint (Figure 1c). The other lateral boundaries were set to free displacement. Before computing the solution, the model was meshed using a physics-controlled tetrahedral mesh, whose parameters are reported in Table 2.

Finally, a stationary study was used to solve the FE model. The results were evaluated in terms of the overall displacement field and deformation, as well as the octahedral shear strain distribution $\hat{\varepsilon }$ (a-dimensional) on the internal scaffold strands, which was computed as follows [49] (Equation (1)):

(1) $$\hat{\varepsilon }=\frac{1}{3}\sqrt{{\left(\varepsilon_{1}-\varepsilon_{2}\right)}^{2}+{\left(\varepsilon_{1}-\varepsilon_{3}\right)}^{2}+{\left(\varepsilon_{2}-\varepsilon_{3}\right)}^{2}}$$

where $\varepsilon_{1}$, $\varepsilon_{2}$, and $\varepsilon_{3}$ represent the principal strain components of the strain tensor.

### 2.3. Scaffolds Fabrication

The polymer blend of PLLA/PCL/PHBV (90/5/5 wt.%) (designated as “blend” from here on) was prepared following an established protocol [47,48]. Briefly, pellets of each individual synthetic polymer were combined, and their mixture was poured into a filament extruder (Rondol Microlab, 10 mm twin screw extruder, Strasbourg, France) to create the thermoplastic filament. Two cycles of extrusion were completed to ensure solution homogeneity and a filament pelletiser (Rondol, Strasbourg, France) was employed to produce the pellets. Afterwards, the resulting pellets were placed back into the extruder hopper and subjected to a second round of extrusion to produce the final printable filament. For the filament production, a 10 mm twin-screw melt extruder Microlab was used connected to a twin belt haul off (Rondol, Strasbourg, France). Laser measurement at the end of the haul off system allowed for a live filament diameter reading to ensure a consistent filament diameter (1.75 ± 0.2 mm) with dimensions optimal for 3D printing.

Scaffolds were printed using FDM (i.e., an additive manufacturing technology which consists of extruding a thermoplastic filament through a nozzle at high temperatures onto a printing plate to create a 3D object layer-by-layer [50], starting from the extruded filaments using a previously described set-up [48]). Briefly, a custom-made bioprinter including (i) a 0.4 mm brass nozzle, (ii) a textured printing plate, and (iii) an enclosure to maintain uniform temperatures during printing was used. The printing parameters, which were previously optimized [48], are summarized in Table 3.

### 2.4. Seeding of MC3T3-E1 onto the Polymeric Scaffolds

Pre-osteoblastic MC3T3-E1 cells (DSMZ Braunschweig, Germany, ACC-210) were isolated from newborn mouse calvaria [51] and were cultured in a humidified incubator at 37 °C and 5% CO_2_ (Heal Force, Shanghai, China) in alpha-MEM medium supplemented with 10% (*v*/*v*) fetal bovine serum (FBS), 100 μg/mL penicillin and streptomycin, 2 mM L-glutamine (all from PAN-Biotech, Aidenbach, Germany), and 2.5 μg/mL amphotericin (Gibco, Thermo Fisher Scientific, Waltham, MA, USA). Cells were harvested from the scaffolds using trypsin-0.25% ethylenediaminetetraacetic acid (EDTA) (Gibco, Thermo Fisher Scientific, Waltham, MA, USA). All of the experiments were conducted with cell passages ranging between P12 and P15. For the mechanical stimulation protocol, each scaffold (5 mm × 5 mm × 1 mm) was submerged for 30 min in complete medium (alpha-MEM) to achieve equilibrium and, therefore, enhance the cell seeding process. After medium aspiration, 7 × 10^4^ cells were seeded onto each scaffold and, after 24 h, they were transferred to new well plates to exclude non-adhered cells on scaffolds. Prior to seeding, all scaffolds were sterilized by immersion in 70% ethanol for 3 min, followed by 30 min of UV irradiation at 265 nm. After 3 days of culture, the medium was replaced with osteogenic medium comprising alpha-MEM supplemented with 10 nM dexamethasone, 10 mM β-glycerophosphate, and 50 μg/mL L-ascorbic acid 2-phosphate, all from Sigma-Aldrich (St. Louis, Missouri, MO, USA).

### 2.5. Mechanical Stimulation Protocol

**Figure 2:**
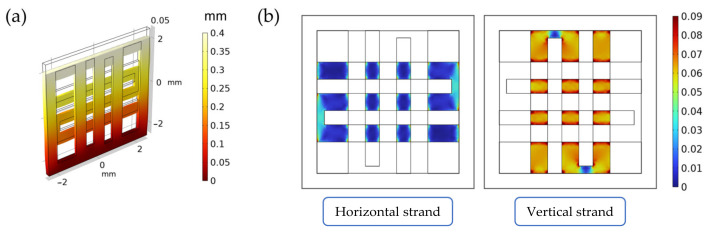
Summary of the results from the FE simulations of the scaffold under mechanical loading. In (**a**), the displacement field inside the scaffold (measured in mm). In (**b**), the octahedral shear strain distribution (a-dimensional) on the horizontal and vertical strands.

A mechanical force was applied to each of the cell-seeded scaffold using a MCTX bioreactor with uniaxial cyclic compression (CellScale, Waterloo, ON, Canada) to allow for a homogeneously distributed applied strain to the cell layer. Cell-loaded polymeric scaffolds were subjected to a confined uniaxial compression (the initial cell loading surface of the scaffolds was turned at 90° for the mechanical stimulation (Figure 2) at three different frequencies of 0.5, 1, and 1.5 Hz and with strain equal to 8% of the scaffold side (i.e., 400 μm of displacement)). Every compression cycle was scheduled for 40 min every day, for 21 days in total. The mechanical stress was applied for the first time the day after the addition of the osteogenic media, named as Day 1 of the experiment. To track differences between stimulated and non-stimulated cells, and to further observe the impact of the mechanical stimulation on cell viability and differentiation, control static cultures were kept under identical conditions but without mechanical stimulation.

### 2.6. Cell Morphology by Scanning Electron Microscopy (SEM)

The cell adhesion and proliferation profiles of the cell-mounted scaffolds were monitored using SEM (JEOL JSM-6390 LV, Tokyo, Japan). Prior to the microscopy, each scaffold was rinsed twice with PBS buffer to remove the remaining culture medium and was subsequently fixed using 4% paraformaldehyde for 15 min. The scaffold was then dried completely using a critical point drier (Baltec CPD 030, Baltec, Los Angeles, CA, USA), then sputter-coated with a gold layer of 20 nm (Baltec SCD 050, Baltec, Los Angeles, CA, USA) and observed using a scanning electron microscope (JEOL JSM-6390 LV, Tokyo, Japan) at 20 kV of accelerating voltage.

### 2.7. Pre-Osteoblastic Cell Viability Evaluation

The PrestoBlue^TM^ assay (Invitrogen Life Technologies, Waltham, MA, USA) was employed to determine the biocompatibility of the scaffolds [52]. It is a resazurin-based reagent, which changes from a blue to a purple color in a reducing environment and is, therefore, indicative of the cell viability. Three different time points were selected to assess the cell growth rate: Day 7, Day 14, and Day 21. The resazurin reduction levels were measured by using a spectrophotometer (Synergy HTX Multi-Mode Microplate Reader, BioTek, Winooski, VT, USA) at 570 and 600 nm. Absorbance values were correlated to cell number using a calibration curve. Samples were analyzed in quadruplicates in three independent experiments (n = 12).

### 2.8. Alkaline Phosphatase Activity Assessment

Alkaline phosphatase activity, an enzyme that is prominent mainly at early osteogenic stages, was measured following a well-described protocol [53]. Briefly, the cell-seeded scaffolds remained in culture for 7, 14, and 21 days using osteogenic medium (alpha-MEM supplemented with 50 μg/mL l-ascorbic acid 2-phosphate, 10 mM dexamethasone, and 10 mM β-glycerophosphate) as described in Section 2.3. At each time point, each scaffold was rinsed twice with PBS and then submerged in 200 μL lysis buffer (0.1% Triton X-100 in 50 mM Tris-HCl pH 10.5) to extract the cell lysate. For the lysis to take place efficiently, each scaffold was subjected to two freeze–thaw cycles, alternating between −20 °C and room temperature. Subsequently, 200 μL of a 2 mg/mL p-nitrophenyl phosphate (pNPP, Sigma, St. Louis, MO, USA) solution in 50 mM Tris-HCl (pH 10.5) and 2 mM MgCl_2_ was added to each sample, and then the well-plate was stored in an incubator at 37 °C for approximately 1 h. The reaction was stopped with the addition of 50 μL 1 M NaOH. Absorbance for p-nitrophenol (pNP), the final product of the reaction, was measured using a spectrophotometer (Synergy HTX Multi-Mode Microplate Reader, BioTek, Winooski, VT, USA) at 405 nm. Absorbance values were correlated to pNP mass by using a calibration curve and were followingly normalized to total cell number. Samples were analyzed in quadruplicates in three independent experiments (n = 12).

### 2.9. Calcium Secretion Measurement

Calcium secretion of the cell-seeded scaffolds was determined by the O-cresol phthalein complexone (CPC) method (BIOLABO, Les Hautes Rives, French) [53]. Calcium mineralization is one of the pivotal regulators for the formation of the extracellular matrix (ECM) and is considered to be a late marker of osteogenesis. Briefly, supernatants were collected, and 10 μL from each were mixed with 100 μL of calcium buffer and 100 μL of calcium dye containing 78 μmol/L O-cresol phthalein complexone. Absorbance of the mixture was measured using a spectrophotometer (Synergy HTX Multi-Mode Microplate Reader, BioTek, Winooski, VT, USA) at 550 nm. Absorbance values were correlated to calcium concentration by using a calibration curve and then normalized to total cell number. Samples were analyzed in quadruplicates in three independent experiments (n = 12).

### 2.10. Collagen Secretion by the Pre-Osteoblasts

Collagen type I is the most abundant component of the organic extracellular matrix of bone tissue and, therefore, its secretion and accumulation is a principal marker of the bone formation process [54]. The quantification of collagen secretion in the culture supernatants was performed using a slightly modified protocol that was previously described and is based on the Sirius Red staining method after 7, 14, and 21 days in culture [55]. Briefly, at three time points, Day 7, Day 14, and Day 21, 25 μL of culture medium was diluted in 75 μL of ultrapure deionized water, mixed with 1 mL 0.1% Direct Red 80 (Sigma, St. Louis, MO, USA) dye in 0.5 M acetic acid, and, finally, incubated for 30 min at room temperature. After the centrifugation of samples at 15,000× *g* for 20 min at 4 °C, the scaffolds were washed with 0.5 M acetic acid to remove the non-bound dye. After the final centrifugation, 1 mL of a 0.5 M NaOH solution was added to extract the collagen bound dye complex, which was then measured in a spectrophotometer (Synergy HTX Multi-Mode Microplate Reader, BioTek, Winooski, VT, USA). The absorbance of 200 μL of each solution was measured at 530 nm and correlated to μg/mL of collagen by using a calibration curve. Samples were analyzed in quadruplicates in three independent experiments (n = 12).

### 2.11. Statistical Analysis

Statistical analysis was performed for the cell viability, the ALP activity, the calcium mineralization, and the collagen secretion assessment using the one-way ANOVA Dunnett’s multi-comparison test in GraphPad Prism version 8 software (GraphPad Software, San Diego, CA, USA). *p*-values indicate statistically significant differences. Single symbols (*) show statistically significant differences with *p* < 0.05, two symbols (**) designate *p* < 0.01, three symbols (***) indicate *p* < 0.001, four symbols (****) indicate *p* < 0.0001, and five symbols (*****) indicate *p* < 0.00001.

## 3. Results

### 3.1. Finite Element Simulations of the Scaffold during Mechanical Compression

The scaffold displacement field and the octahedral shear strain distribution computed in the FE model can be seen in Figure 2a. As can be seen from Figure 2b, the vertical strand (i.e., the strand along the displacement direction) supports most of the strain when compared to the horizontal one (i.e., the strand perpendicular to the displacement direction). As specified in Supplementary Materials—S1, this validates the scaffold design and the loading direction, as the seeded cells will be subjected to significant levels of strain during stimulation.

### 3.2. Pre-Osteoblastic Cell Morphology

**Figure 3:**
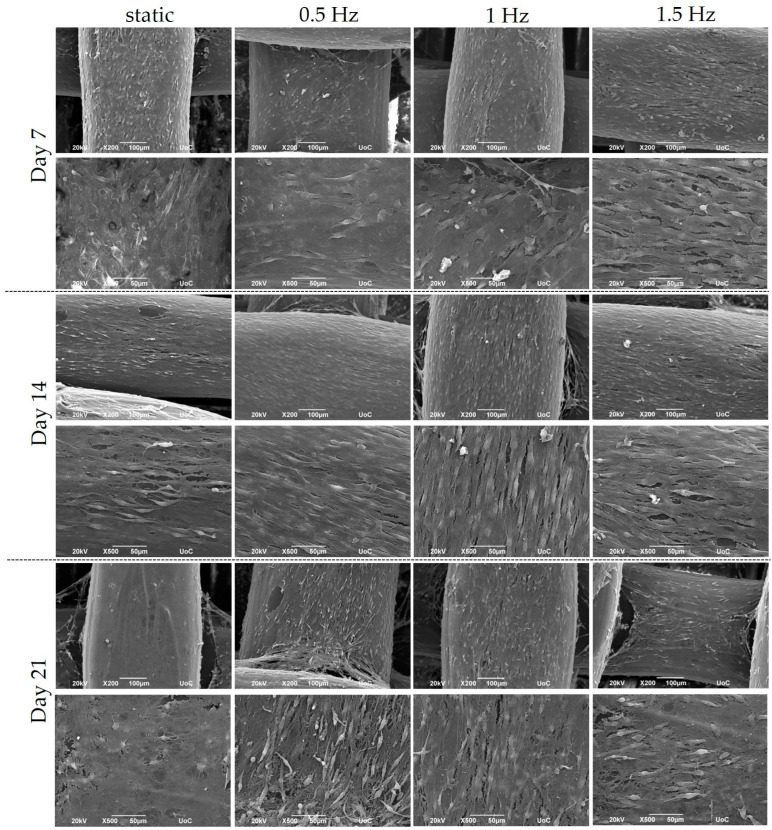
Representative SEM images display the adhesion and morphology of pre-osteoblastic cells within polymeric scaffolds for 7, 14, and 21 days of culture under static and dynamic conditions. All scaffolds present a strong cell adhesion regardless of the examined condition. Original magnification is 200× and 500× at each time point, and scale bars represent 100 μm (upper panel) and 50 μm (lower panel), respectively.

The cubic blends were loaded with pre-osteoblastic cells, and their morphology was visualized using SEM at Days 7, 14, and 21 (Figure 3). The cells appeared to have adhered sufficiently to the scaffolds by Day 7, while also demonstrating the physiological cellular network and the distinctive elongated shape, with an increasing proliferation rate up to Day 21.

### 3.3. Cell Viability Assessment within the Scaffolds

**Figure 4:**
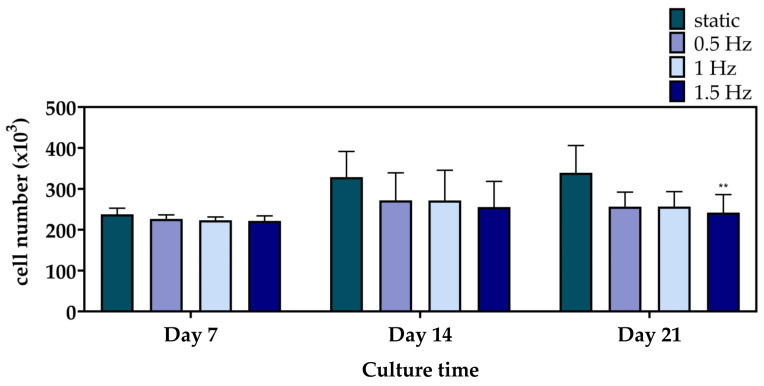
Cell proliferation of the polymeric scaffolds presenting the total number of pre-osteoblastic cells after 7, 14, and 21 days of culture (** *p* < 0.01).

Figure 4 displays the cell viability results of the mechanically stimulated and non-stimulated constructs after 7, 14, and 21 days. At Day 7 and 14, the three dynamic conditions presented a similar cell growth rate to the static culture. Notably, at Day 21, only the 1.5 Hz condition exhibited a significantly lower cell viability, with the 0.5 and 1 Hz conditions still retaining comparable levels.

### 3.4. Evaluation of the Differentiation Potential of the Cell-Seeded Scaffolds

**Figure 5:**
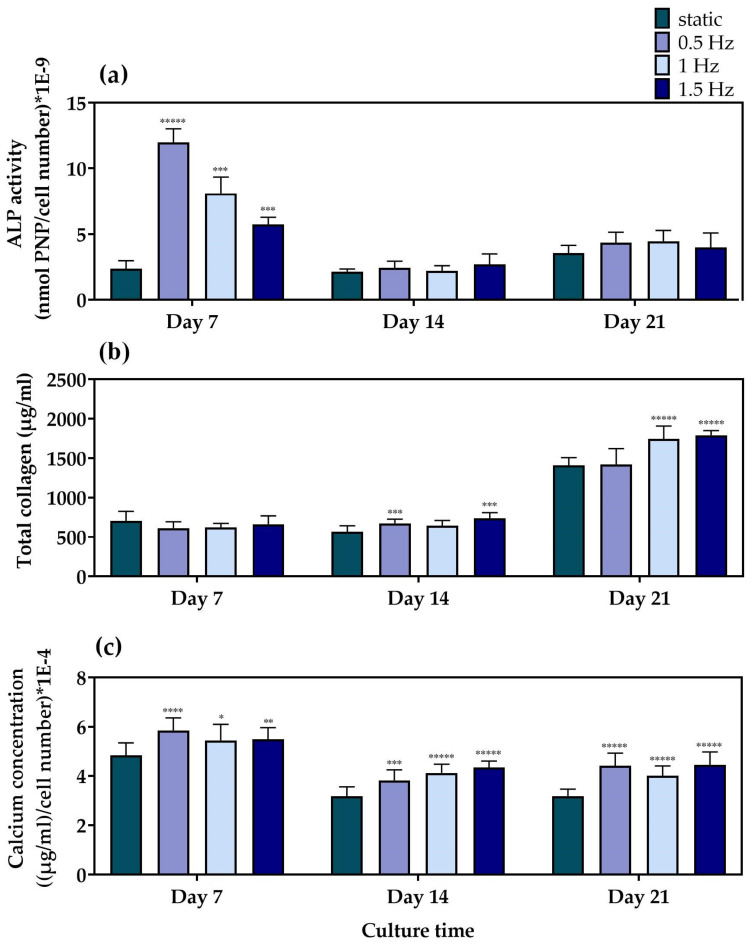
(**a**) ALP activity of the MC3T3-E1 cells loaded onto the polymeric scaffolds after 7, 14, and 21 days normalized to cell number, (**b**) total secreted collagen concentration after 7, 14, and 21 days in culture, and (**c**) calcium production by the pre-osteoblastic cells loaded onto the polymeric scaffolds after 7, 14, and 21 days normalized to cell number (* *p* < 0.05, ** *p* < 0.01, *** *p* < 0.001, **** *p* < 0.0001, ***** *p* < 0.00001).

For the evaluation of the differentiation potential of the mechanically stimulated cell-seeded scaffolds, we examined the alkaline phosphatase activity, collagen concentration, and calcium deposition as representative markers of osteogenesis, after three time points of 7, 14, and 21 days in culture. Alkaline phosphatase (Figure 5a) activity is one of the most representative markers for the early phase of osteogenesis. The main role of this enzyme is to cleave the phosphate groups off different molecules and, therefore, to make them available so that they can be used for the formation of hydroxyapatite crystals, a principal component of the extracellular matrix of bone tissue [56]. Among the different dynamic culture conditions, at Day 7, the condition at 0.5 Hz demonstrated significantly higher ALP activity, followed by the conditions at 1 Hz and the 1.5 Hz, respectively. Moreover, all of the dynamic culture conditions were superior compared to the static control at this particular time point. At Day 14 and Day 21, no statistically significant differences could be observed between the different conditions. In addition, a steep reduction after Day 7 was clearly evident for all conditions, apart from the static culture condition, which retained comparable enzyme activity levels at every time point.

Collagen concentration was quantified in the supernatants as a middle marker of osteogenesis. Collagen type I is a vital constituent and the most abundant organic component of bone tissue that serves a structural role in the ECM formation [57]. The quantification of collagen concentration (Figure 5b) revealed that, at Day 7, the scaffolds depicted comparable collagen production levels under both dynamic and static culture conditions. A slight increase in collagen production was observed after 14 days in culture for the conditions at 0.5 and 1.5 Hz, respectively, compared to the static culture. By Day 21, all three dynamic conditions indicated higher levels than the static equivalent, with the conditions at 1 and 1.5 Hz retaining the highest values.

Calcium deposition (Figure 5c) is considered to be a late marker for osteogenesis. The bone mineralization process requires calcium ions to occur, which is one of the main constituents of the hydroxyapatite structure [58]. At Day 7, all of the dynamic culture conditions demonstrated higher calcium concentration when compared to the static culture, with the values at 0.5 Hz slightly exceeding those at 1 Hz and 1.5 Hz. At Day 14, a decrease in the calcium secretion levels was detected for all conditions compared to Day 7, with all three dynamic conditions displaying higher values compared to the static culture condition. Similar data were obtained on Day 21, without significant differences among the dynamic culture conditions investigated.

## 4. Discussion

Natural bone is a tissue with a high metabolic profile, whose balance is governed by the biomechanical signals that osteocytes exchange. The complexity of the native bone is affected by the application of various mechanical stimuli that the body experiences on an everyday basis. This is compliant with the fact that bone tissue is piezoelectric [59] due to the highly oriented structure of collagen (polar hexagonal crystalline unit) and collagen’s ability to respond to mechanical loading [60]; thus, mechanotransduction pathways play a pivotal role in its development [41,61]. Mechanical stimuli for bone include stretching, compressive stress, and fluid shear stress. Over the years, different research groups have examined the impacts of these stimuli as potential osteogenic stimulants [62,63,64], with the most thoroughly investigated being the cyclic stretching and fluid shear flow [19]. Although there are plenty of studies that have already investigated the effect of mechanical stimuli on cell fate under compressive mechanical loading [65,66,67], most of the time there is no correlation between the examined parameters; therefore, it is difficult to deduce the role of each one individually. In particular, studies comparing the influence of cycle number and frequency on bone tissue formation are scarce in the literature [45,68]. To further explore this area, we used a bioreactor that applies uniaxial compression on the surface of polymer-based, FDM-printed scaffolds and biologically evaluated the cell proliferation and osteogenic potential of pre-osteoblastic cells, which were loaded onto the scaffolds. The dynamic conditions were selected based on the physiologically applied forces during human body movement, and the fact that cells exhibit increased sensitivity to mechanical stimuli at frequencies below 2 Hz [27]. The applied strain was selected based on a previous study, in which MC3T3-E1 cells were seeded on PCL/PLLA-based scaffolds and mechanically stimulated at a frequency of 1 Hz and a cyclic strain of 10% for 20 min daily, which resulted in increased extracellular matrix formation [69]. On that note, we prepared PLLA/PCL/PHBV (90/5/5 wt.%) polymeric scaffolds and employed three dynamic conditions, being 0.5, 1, and 1.5 Hz. Cell-loaded scaffolds in static cultures were used as control. Simulations using FE analysis validated the design of the scaffolds and the loading direction, assuring that the cells cultured within the scaffolds experienced significant levels of strain during mechanical stimulation. At Day 7, all of the conditions exhibited an increase in cell population compared to the initial cell number seeded onto the scaffolds. Between the various conditions, no significant variations in cell viability were detected, except for at day 21, at which the 1.5 Hz mechanical loading led to a decreased cell number compared to the static control. It is interesting to note that the examined dynamic culture conditions did not impair cell proliferation. These findings are strongly supported by the SEM images, which revealed an excellent cell adhesion profile of the pre-osteoblasts by Day 7, regardless of the condition, while at both Day 14 and 21, the scaffolds were evidently covered with dense cell sheets. The differentiation potential of the applied stress was measured by the determination of two of the most indicative osteogenic markers, the ALP activity and the calcium mineralization. At Day 7, the ALP activity of all three of the dynamic conditions demonstrated significantly higher levels than those of the static culture, with the condition at 0.5 Hz retaining the highest value, followed by those at 1 Hz and 1.5 Hz, respectively. However, at Day 14 and 21, the enzyme activity in all of the dynamic conditions was significantly downregulated, as the maturation towards osteoblasts had already occurred by Day 7. The collagen production profiles showed similar trends for the first two time points, with the conditions of 1 Hz and 1.5 Hz presenting the highest values at day 21, illustrating that the formation of the extracellular matrix was more prominent at this time point. Similarly to the ALP activity, the calcium secretion levels also peaked at Day 7, with the dynamic conditions surpassing the static condition and the 0.5 Hz condition having the optimal response. Although a decline in calcium production was evident at Day 14 and 21, for all conditions, the mechanically stimulated scaffolds depicted significantly higher values compared to the static scaffolds at both time points. Previous studies have shown that the application of cyclic uniaxial compression at 1 Hz on mesenchymal stem cells enhanced collagen type I, osteonectin expression, and calcium production after 14 and 21 days in culture, respectively [70]. Buckley et al. also focused on the effects of mechanical stimulation on pre-osteoblastic cells, reporting increased levels of ALP activity within 48 h, and a significant increase in collagen production after 72 h [71]. Another study reported on the application of cyclic stretching to human bone-derived cells, with a frequency of 1 Hz, which revealed an enhancement in cell proliferation and collagen production. Conversely, the expression of alkaline phosphatase and osteocalcin was downregulated, suggesting that the cyclic stretching stimulation favored the matrix production processes over osteogenic differentiation [72]. Moreover, the application of cyclic compression of 0.5 Hz [73] and 1 Hz [74] on mesenchymal stromal cells has also been correlated to decreased cell proliferation capacity, while the 0.5 Hz frequency led to overexpression of RUNX2 and osteocalcin, two bone related markers. Surprisingly, the decrease in cell proliferation at the 1 Hz condition did not increase the cell differentiation, as the expression of these two markers was downregulated [74]. Mechanical loading protocols have been employed as biomimicking models to simulate the physiological tooth chewing movement. The application of 1 Hz frequency for 30 min daily on human dental pulp stromal cells seeded into 3% w/v agarose gel led to an amplification of the ALP activity, and calcium and collagen deposition after seven days in culture compared to the static cultures, illustrating the importance of teeth mobility in the formation of bone tissue [75]. Overall, we proposed a cost-effective dynamic ex vivo model to examine the effect of uniaxial compression on the osteogenic capacity of cell-laden 3D printed scaffolds. We demonstrated that all three of the examined frequency conditions significantly promoted osteogenesis compared to the static control cultures, without compromising the cell viability during compression. Our dynamic model could be combined with other forces that control the growth of bone tissue, including shear stress. Such combinations could recreate the physiological in vivo situation, broadening the exploitation of biological results by minimizing the use of animal studies. Furthermore, our model can be utilized for the investigation of even more complex in vitro biological systems such as co-cultures to underline how mechanical stimulation affects the cross-talk between various cell populations and the unraveling of molecular pathways regarding osteogenic and osteoclastogenic cell differentiation [3].

## 5. Conclusions

In this study, we examined the impact of three frequency values of cyclic uniaxial compression on the osteogenic response of constructs comprising pre-osteoblastic cell-laden 3D printed PLLA/PCL/PHBV polymer blend scaffolds. The constructs were mechanically stimulated every day for 40 min at a displacement of 400 μm at 0.5, 1, and 1.5 Hz for 21 days. Finite element simulation was performed to validate the scaffold design, the loading direction, and the stimulation of cells with significant strain values. The pre-osteoblastic cell behavior was monitored by measurement of their cell viability and osteogenic maturation over a period of 21 days. The dynamic culture conditions did not affect the cell viability and proliferation. The osteogenic differentiation of the pre-osteoblasts under dynamic culture conditions was significantly increased compared to the static control conditions, as evidenced from the ALP activity, and collagen and calcium production data. Moreover, the ALP activity and calcium production results revealed the highest osteoinductive effect at 0.5 Hz.

## Figures and Tables

**Table 1:** Summary of the material properties used during the FE simulations. The values correspond to those previously reported in [47].

Material Property	Symbol	Unit of Measure	Value
Young modulus	*E*	MPa	32.2
Poisson ratio	*N*	-	0.3
Density	*Ρ*	kg/m^3^	1200

**Table 2:** Summary of the mesh properties for the solid mechanics model.

Mesh Setting	Average Size [mm]	Min Size [mm]	Max Size [mm]	Model DoF
Finer	0.23	0.10	0.34	≈42 k

**Table 3:** Summary of the main printing parameters used to produce the scaffolds for the mechanical loading experiments.

Printing Speed [mm/s]	Extrusion Temperature [°C]	Bed Temperature [°C]	Layer Height [mm]
10	210	80	0.2

## Data Availability

The data presented in this study are openly available in ZENODO: https://doi.org/10.5281/zenodo.7244377 (accessed on 24 October 2022).

## Supplementary Materials

The following supporting information can be downloaded at: https://www.mdpi.com/article/10.3390/bioengineering10050532/s1, Figure S1: In (a), overview of the cylindrical scaffold CAD model, while in (b) summary of the boundary conditions used in the Comsol model; Table S1: Summary of the mesh settings for the cylindrical scaffold mechanical simulations; Figure S2: Summary of the displacement field and octahedral shear strain distribution inside the cylindrical scaffold.
