# Supplementary material for: Effect of Uniaxial Compression Frequency on Osteogenic Cell Responses in Dynamic 3D Cultures
Source: Bioengineering (Basel). 2023 Apr 27;10(5):532. doi: 10.3390/bioengineering10050532 (PMC10215508; doi:10.3390/bioengineering10050532)
Supplement: Supplementary file 1 [file bioengineering-10-00532-s001.zip › bioengineering-2306293-supplementary.docx]

Supplementary Materials

Effect of Uniaxial Compression Frequency on Osteogenic Cell Responses in Dynamic 3D Cultures

Georgia-Ioanna Kontogianni ^1^, Konstantinos Loukelis ^1^, Amedeo Franco Bonatti ^2^, Elisa Batoni ^2^, Carmelo De Maria ^2^, Raasti Naseem ^3^, Kenneth Dalgarno ^3^, Giovanni Vozzi ^2^, David B. MacManus ^4^, Subrata Mondal ^4^, Nicholas Dunne ^4^, Chiara Vitale-Brovarone ^5^ and Maria Chatzinikolaidou ^1,6^

1. Section S1 – details on the scaffold design procedure

The overall CAD geometry of the scaffold is reported in Figure S1.1a. As can be seen from the figure, the scaffold consists of a cylindrical shape of 5 mm diameter and 1 mm height, with an internal porosity of 50% using a line infill. A bottom layer was included to avoid cell slippage from the scaffold during seeding. The scaffold CAD geometry was imported in Comsol Multiphysics and a Finite Element model was set up using the Solid Mechanics module. The material properties are reported in Table 1, while the boundary conditions set for the model can be seen in Figure S1.1b. A prescribed displacement of 400 µm was set as described in the manuscript.


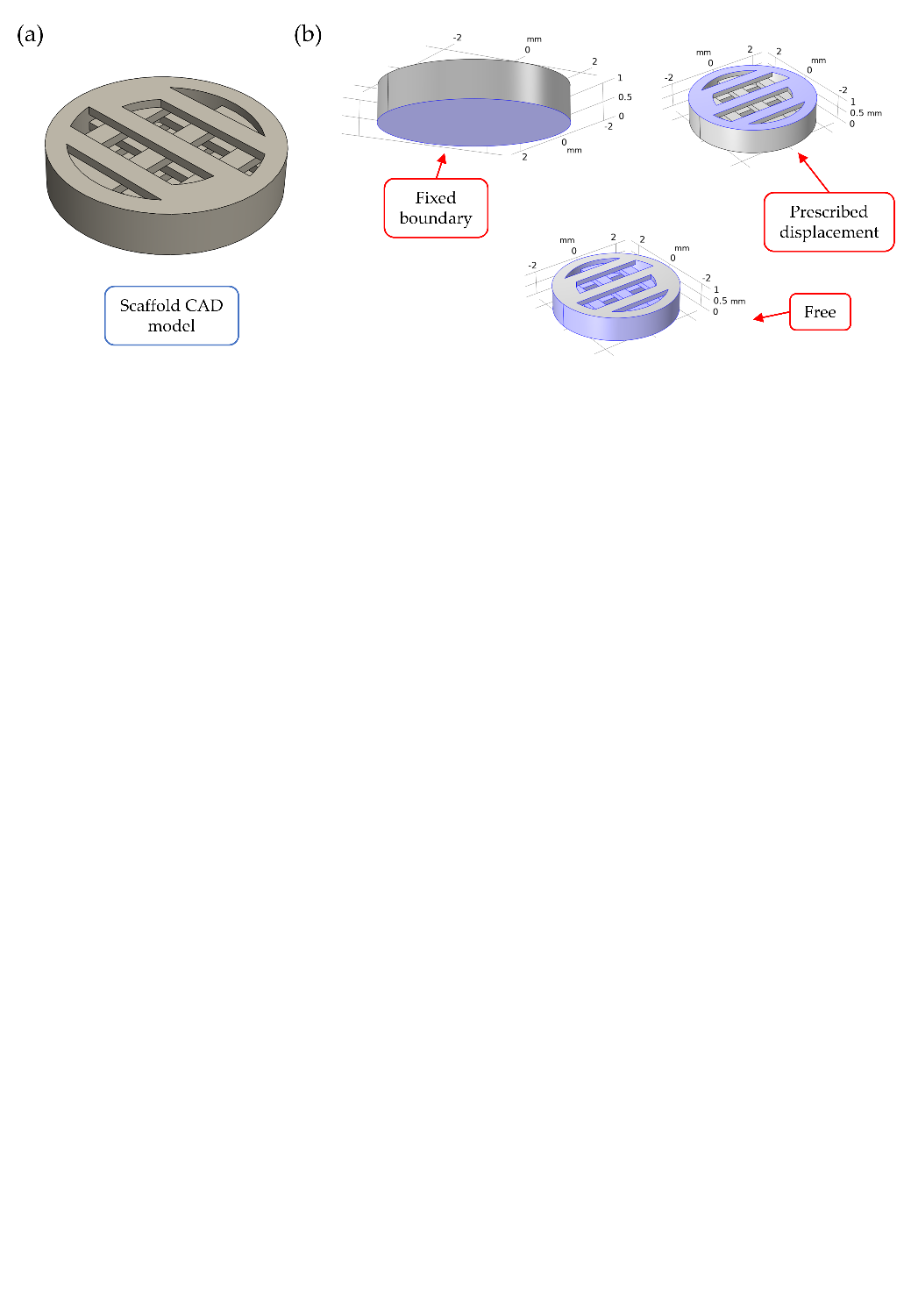


**Figure S1.** (**a**) Overview of the cylindrical scaffold CAD model, (**b**) summary of the boundary conditions used in the Comsol model.

Before computing the solution, the model was meshed using a physics-controlled tetrahedral mesh, whose parameters are reported in Table S1.1.

**Table S1.** Summary of the mesh settings for the cylindrical scaffold mechanical simulations.

| **Mesh setting** | **Average size [mm]** | **Min size [mm]** | **Max size [mm]** | **Model DoF** |
| --- | --- | --- | --- | --- |
| Finer | 0.28 | 0.04 | 0.41 | ≈109k |

Finally, a stationary study was used to solve the FE model. The results were evaluated in terms of the overall displacement field and deformation, as well as the octahedral shear strain distribution as described in the manuscript. The shear strain distribution was evaluated over the internal scaffold strands as specified in the manuscript.


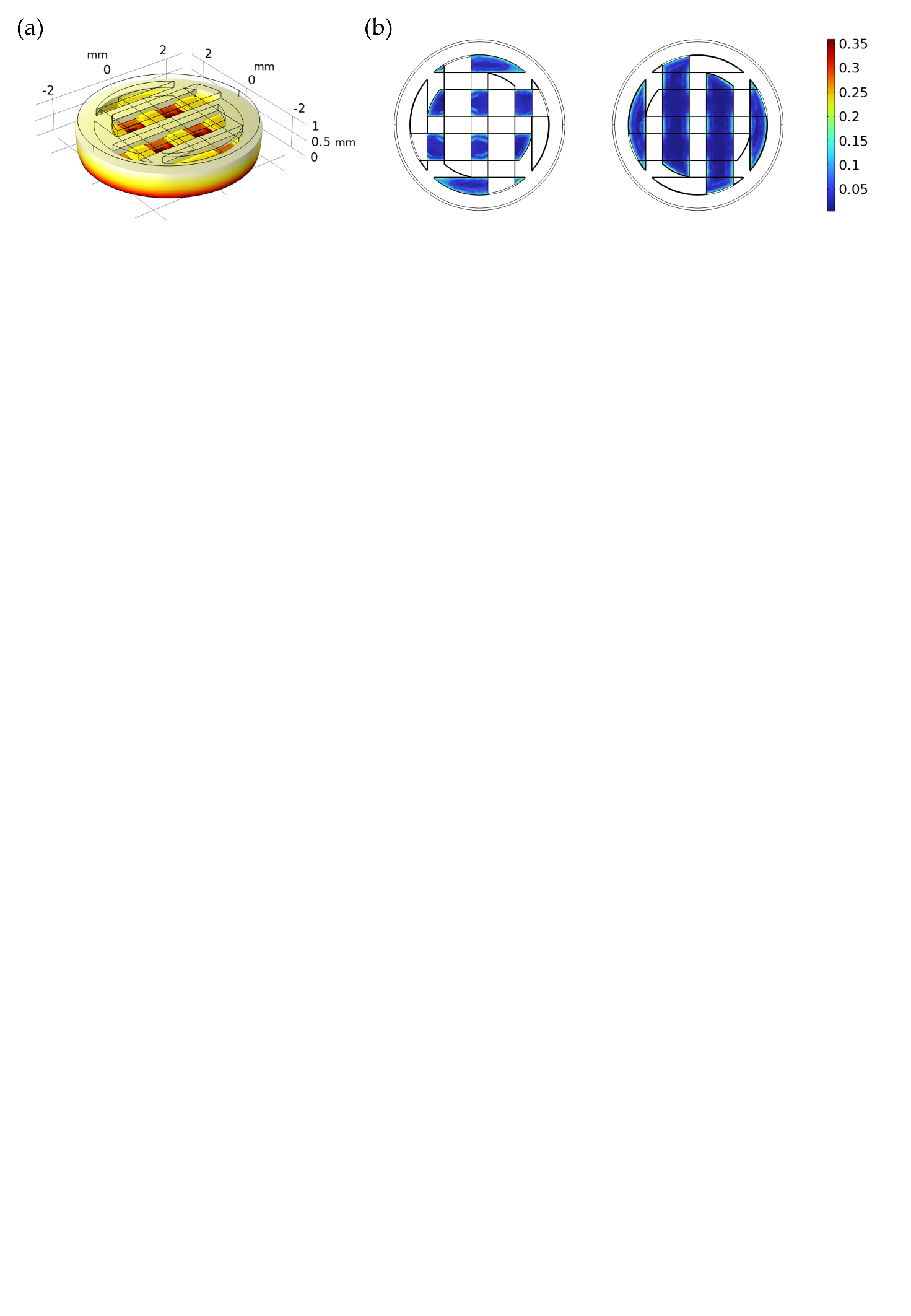


**Figure S2**. Summary of the displacement field and octahedral shear strain distribution inside the cylindrical scaffold.

As can be seen in Figure S1.2b, the octahedral shear strain is close to zero over all of the evaluated lines. As a result, seeded cells on this scaffold geometry would experience a negligible strain; thus, this scaffold geometry was not used for printing and biological evaluation experiments.
